# Development of Virtual Metrology Using Plasma Information Variables to Predict Si Etch Profile Processed by SF_6_/O_2_/Ar Capacitively Coupled Plasma

**DOI:** 10.3390/ma14113005

**Published:** 2021-06-01

**Authors:** Ji-Won Kwon, Sangwon Ryu, Jihoon Park, Haneul Lee, Yunchang Jang, Seolhye Park, Gon-Ho Kim

**Affiliations:** 1 Department of Energy Systems Engineering, Seoul National University, Seoul 08826, Korea; anakin1229@snu.ac.kr (J.-W.K.); swryus@snu.ac.kr (S.R.); wlgnstlsqkf@snu.ac.kr (J.P.); haneul7475@snu.ac.kr (H.L.); js1wofl@snu.ac.kr (Y.J.); 2 Samsung Display Co. Ltd, Samsung-ro, Tangjeong-myeon, Asan-si 31454, Korea; druyvesteyndf@gmail.com

**Keywords:** virtual metrology, plasma information, etch profile, real-time etch depth, bowing CD, rectangular etch profile model, etch area, etch plasma, process monitoring technology

## Abstract

In the semiconductor etch process, as the critical dimension (CD) decreases and the difficulty of the process control increases, in-situ and real-time etch profile monitoring becomes important. It leads to the development of virtual metrology (VM) technology, one of the measurement and inspection (MI) technology that predicts the etch profile during the process. Recently, VM to predict the etch depth using plasma information (PI) variables and the etch process data based on the statistical regression method had been developed and demonstrated high performance. In this study, VM using PI variables, named PI-VM, was extended to monitor the etch profile and investigated the role of PI variables and features of PI-VM. PI variables are obtained through analysis on optical emission spectrum data. The features in PI-VM are investigated in terms of plasma physics and etch kinetics. The PI-VM is developed to monitor the etch depth, bowing CD, etch depth times bowing CD (rectangular model), and etch area model (non-rectangular model). PI-VM for etch depth and bowing CD showed high prediction accuracy of R-square value (R^2^) 0.8 or higher. The rectangular and non-rectangular etch area model PI-VM showed prediction accuracy R^2^ of 0.78 and 0.49, respectively. The first trial of virtual metrology to monitor the etch profile will contribute to the development of the etch profile control technology.

## 1. Introduction

The plasma enhanced etch process using fluorine compounds such as CF_4_, NF_3_, and SF_6_, is one of the most important process in the semiconductor fabrication, which requires etch profile controls of nanoscale critical dimension (CD) and high aspect ratio. The increase in difficulty of the etch profile control is due to the decrease of critical dimension (CD) and increase of aspect ratio [1]. In addition, the measurement and inspection (MI) process to manage the etch profile is essential, but it takes a long time and reduces the manufacturing throughput. It has limitations in a real-time monitoring during the process [2]. Alternatively, virtual metrology (VM) was developed to overcome the limitations of MI. It is an algorithm that predicts the process results by statistically processing data of EES, sensor, and preceded process MI [3]. Here, EES represents the equipment engineering system data gathered from equipment parts involved in the semiconductor process chamber such as mass flow controller (MFC), RF power, matcher, and throttle valve potentiometer (TVP). Sensor data refer to data acquired from the optical emission spectroscopy (OES) and voltage-current (VI) probe. It is known that the process results such as etch rate, deposition rate, and fault occurrence can be predicted using the data gathered during the process [4,5,6,7,8]. VM is basically a data process algorithm so it has the advantage of being able to check the process result in real-time during the process. Compared to the conventional MI, the runtime to check the process result is much shorter, and the analytic information of the process result can be obtained at a relatively low cost. However, the VM has a limitation in prediction accuracy. Many studies are being conducted to improve the accuracy, and most of them are being developed through choosing more sophistical statistical methods and utilizing more sensor data.

J. M. Gu et al., (2015) developed VM model that predicts the TSV etch depth of 80 and 25 μm in diameter based on the partial-least-square regression (PLSR) method and multilayer feedforward neural networks with back-propagation learning algorithm for OES data [9]. It was shown that it was possible to predict the etch depth of TSV with the mean-square-error (MSE) of 0.08 μm and the maximum error of 0.422 μm for the 80 μm diameter TSV. S. Lynn et al., (2010) developed the etch rate VM algorithm in the five-step industrial trench etch process [10]. The statistical analysis was performed with the weighted window PLS model using 90 different variables such as time series measurements of power delivered to the chamber, matchbox inductor positions, internal chamber temperatures, gas flow rates, and end point times. The etch rate was predictable at the level of MSE 0.255 and R-square value (R^2^) of 0.734. These VM models demonstrated high accuracy and robustness. In addition, the prediction accuracy of VM was improved with the increased number of input variables and more complicated algorithm.

Alternatively, a new approach is carried out to develop the high prediction accuracy VM model which has reduced the number of input data with a basic statistical method and simplified the development procedure of VM. Recently, it was shown that using novel parameters describing characteristics of the process plasma, called plasma information (PI) variables as input data of VM improved the prediction accuracy. For example, the etch rate is determined by the characteristics of the etch process plasma. Here, the etch process plasma is composed of electrons, ions, and neutral (including radical) species. Plasma characteristics include electron density, electron temperature, ion density, and neutral species density in which the production is closely coupled with the ionization reaction and the dissociation reaction. Those ions and neutral etchant species participate in etch process, and the etch rate is, specifically, proportional to the energy and flux of etchant [11]. It implies that the etch process is carried out by plasma-chemical reaction in etch process plasma. In previous studies, the factorized values of these plasma characteristics, called PI variables, are adopted in the development of the VM model. A virtual metrology using PI variables, which is abbreviated as PI-VM, was demonstrated that it has a high accuracy.

H. J. Roh et al., (2018) developed the nitride film thickness PI-VM for the nitride-oxide PECVD process in capacitively coupled plasma (CCP). The residue accumulation on the chamber wall was defined as PI_Wall_, and the electron density and temperature were defined as PI_Bulk_ [4]. Each was measured based on the data obtained from the OES sensor. Using PI_Wall_ and PI_Bulk_ as the input data of VM, it was demonstrated that the prediction accuracy for nitride thickness increased and the mean-absolute-percentage-error (MAPE) decreased from 0.59% to 0.33%. S. Park et al., (2015) and Y. C. Jang et al., (2019) developed PI-VM model that predicts the etch rate and etch depth with the electron energy distribution function (EEDF) [12,13]. The PI_EEDF_ variable (b-factor) which represents the shape of the EEDF changes the reaction rates of dissociation and ionization in CCP of the SiO_2_ trench etch process. Developed PI-VM model reveals that PI_EEDF_ is sensitive to the variation of passivation and etch rate. In the sense of the statistical method, S. Park used the principal-component-analysis (PCA) method, and Y. C. Jang evaluated the PI-VM developed with various statistical methods of PCA, Pearson-correlation-filter (PCF), stepwise-variable-selection (SVS), and Tatsumi’s method. Compared to the conventional VM, PI-VM using PI_EEDF_ with various statistical methods showed higher R^2^ and lower MAPE. This clarified that using PI can develop a more accurate and higher performance of the VM model.

On the other hand, in terms of etch profile monitoring, tracing the horizontal etch becomes important as the vertical etch, especially with the shrinking CD. K. Nomura et al., (2011) developed the critical dimension (CD) VM model for the SiO_2_ contact hole etch process in parallel-plate dual-frequency CCP [14]. CD was predicted by the statistical analysis of 20 EES data and OES spectrum data using the PLS with a population renewal method. Furthermore, H. K. Lee et al., (2017) introduced the wafer-to-wafer control algorithm of the difference between the edge CD and center CD using the developed VM [15]. As the etch process progressed, center holes were eroded and the difference between the edge CD and the center CD increased. VM for difference between edge CD and center CD was developed by the PLS method using EES data, OES data, and VI-probe data and it showed R^2^ of 0.89. By difference between edge CD and center CD VM and control of the center weighting (the gas flow rate between the center and edge areas in a showerhead), the standard deviation of the CD was reduced by nearly 40%.

D. Kim et al., (2007) simulated the etch profile in the etch process of the SiO_2_ layer with the developed photo-resistor (PR) [16]. The particle-based Monte-Carlo algorithm and analytic ray tracing algorithm for the transport of ions and neutrals were applied to the profile simulator, respectively. The angle of primary and secondary facets, top CD, neck CD, and bowing CD can be obtained from the profile simulator. In the case of simulation, it is useful to obtain various etch profile information and to evaluate the etch profile variation caused by the process recipe and plasma characteristics. However, it is difficult to derive the etch profile information in a short time due to the requirement of high computing power. Although the importance of etch profile management has been grown, there is a lack of capable VM to monitor the etch profile including both the vertical and the lateral etch rate.

In this study, PI-VM models are developed to predict the etch profile in terms of etch depth, bowing CD, etch depth times bowing CD (ED × BCD, rectangular model), and etch area (non-rectangular model) using the data of EES, sensor, and PI variables with statistical analysis method of multi-linear regression-stepwise variable selection (MLR-SVS). In the process of developing PI-VM, the contribution of PI to the accuracy improvement is evaluated, and the features selected among the input data are analyzed from the aspect of plasma physics and etch kinetics. Finally, the role of features and the prediction accuracy of four of the etch profile PI-VM are investigated.

This paper is organized as follows: In Section 2, the experimental setup, the development procedure of PI-VM, the method to obtain PI, and the definition of four etch profile quantified factors (etch depth, bowing CD, ED × BCD, and etch area) are introduced. In Section 3, the effect of PI on the prediction accuracy and the details of PI-VM models for the four etch profile values are explained. In Section 4, the conclusions are provided.

## 2. Development of Etch Profile PI-VM

### 2.1. Experimental Setup

**Figure 1:**
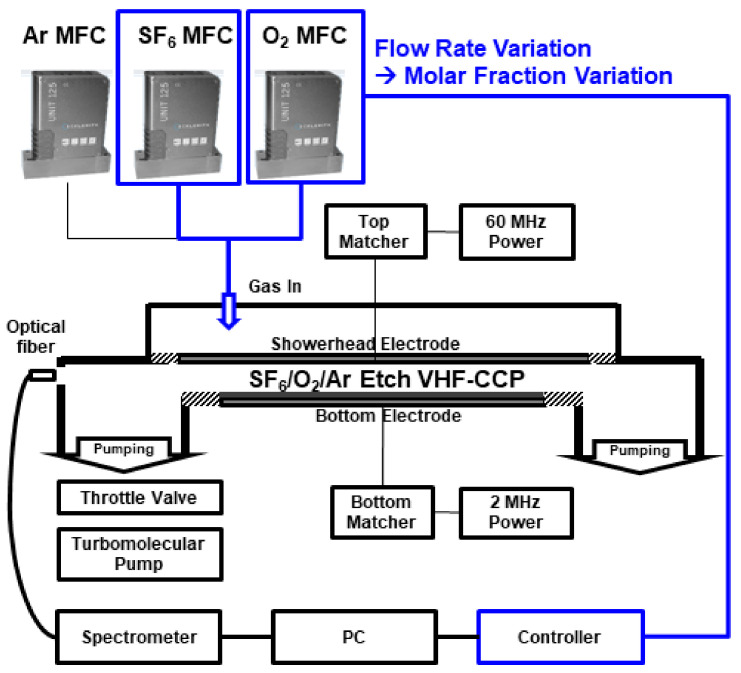
Schematic diagram of the CCP chamber used for Si etching. SF_6_, O_2_, and Ar are delivered through the MFC, and the pressure inside the chamber is maintained at 20 mTorr by the throttle valve. The VHF power of 60 MHz is delivered to the top electrode, and the LF power of 2 MHz is delivered to the bottom electrode. The emitted light of the plasma is observed through the OES sensor in which the viewport is located on the outer wall of the chamber.

Figure 1 shows a schematic diagram of the Si etching chamber used to acquire the data for etch profile PI-VM development, and the etch process conditions are summarized in Table 1. SF_6_, O_2_, and Ar gas flows were controlled by each MFC and the mixture gas was delivered through showerhead of top electrode. The throttle valve is controlled to maintain the operating pressure in the chamber at 20 mTorr. The Ar flow rate was fixed at 50 sccm while the flow rate of SF_6_ and O_2_ was varied to change the molar ratio. The flow rate of SF_6_ was changed from 96 to 144 sccm, and the flow rate of O_2_ was changed from 60 to 90 sccm. RF powers of 500 W of 60 MHz VHF (very-high frequency) and 150 W of 2 MHz LF (low frequency) are applied to the top (main power) and bottom electrodes (bias power), respectively. The diameter of the bottom electrode is 300 mm and the inner diameter of the wall is 600 mm. The gap distance between the top and bottom electrode is 22.5 mm. Assuming the plasma is generated with a cylinder shape between the two electrodes in the inner wall of the chamber, the volume is π × 0.3 × 0.3 × 0.0225 = 6.36 × 10^−3^ m^3^. The electron density diagnosed with the Langmuir probe showed an order of 10^10^ cm^−3^ at the center. The tungsten probe tip with a diameter of 0.3 mm and a length of 3 mm was used. The optical emission spectrum from the plasma is observed by the OES Sensor (Avaspec ULS-2048L 2 channel spectrometer, Avantes, Netherlands) of which the view-port is located at the outer wall. The spectrometer slit size is 25 μm. Channels 1 and 2 acquire optical signals in the 250–500 nm wavelength band and 500–1000 nm wavelength band, respectively. The pixel resolution of channels 1 and 2 are 0.13 and 0.26 nm, respectively. The optical resolutions, which mean full-width half maximum (FWHM) of atomic optical emission signal, are 0.3 and 0.5 nm for channels 1 and 2, respectively. After passing through the quartz and optical fiber of the chamber outer wall, the optical emission signal reaches the spectrometer. In order to calibrate the sensitivity of spectrometer according to the wavelength, the Avalight-DH-Cal halogen source is used as a standard light source [17]. The OES sensor acquires the spatially averaged optical signal of the plasma generated at the interspace between two electrodes, and it is assumed that the spatial distribution of the plasma is uniform. The radiation-trapping effect is not considered in the analysis of OES data.

### 2.2. Flow Chart of PI-VM Model Development

**Figure 2:**
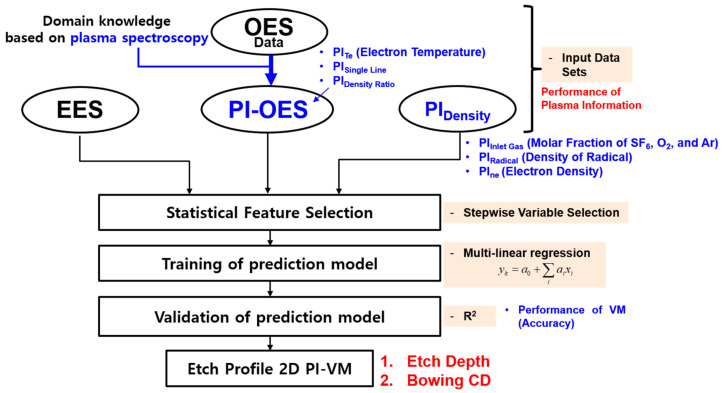
Flow chart of etch profile PI-VM model development.

Figure 2 shows a flow chart of the etch profile PI-VM model development [13]. Data acquired during the etch process and PI variables compose the input data sets. It goes through the process of statistical feature selection, training/validation of etch profile VM. Four quantified values of defining etch profile will be discussed in a later section. The data acquired during the process are classified into three categories: EES, PI-OES, and PI_Density_. The EES data are acquired from the etch process chamber parts such as the MFC flow rate, TVP position, and matcher cap position. PI-OES does not include all the data acquired from 2048 pixels of spectrometer, and uses the intensities and line ratios of the emission lines which are selected by domain knowledge based on the plasma spectroscopy. PI-OES is divided into PI_Te_, PI_Single Line_, and PI_Density Ratio_. PI_Te_ represents the electron temperature of the plasma. PI_Single Line_ and PI_Density Ratio_ represent the single line emission intensity and line intensity ratio between specific species, respectively. PI_Density_ is divided into PI_Inlet Gas_, PI_Radical_, and PI_ne_. PI_Inlet Gas_ represents the density of SF_6_, O_2_, and Ar which is the gas species flow into the chamber through the MFC. Here, PI_ne_ means electron density, and PI_Radical_ is the densities of radical which is analyzed with the electron-impact dissociation reaction of the etch process plasma. The variables and descriptions are summarized in Table 2.

By using the statistical method, the features of high correlation with the etch profile are selected. The process of selecting the features among the input data set is called statistical feature selection, and the stepwise variable selection method is applied in this study. The stepwise variable selection (SVS) is a method of sequentially selecting high correlation variables based on a pre-specified criterion such as F-test and *t*-test. The multi-linear regression (MLR) model, which is a method of expressing the prediction target by a linear combination of selected features, is used as a prediction model [18]. Finally, the evaluation on the prediction accuracy of the developed VM model is carried out with the R-square value (R^2^). When R^2^ is closer to 1, it implies a higher accuracy of prediction.

### 2.3. Description of Plasma Information Variables from OES Data

PI variables are obtained from the analysis of sensor data. The accuracy of the PI variables is compared with the diagnostic results and it ensured to trace a drift of the plasma characteristics. The PI variables obtained by OES are named PI-OES. The method to generate PI data from sensor data is described in detail in the later section and previously published papers [4,12,13].

**Figure 3:**
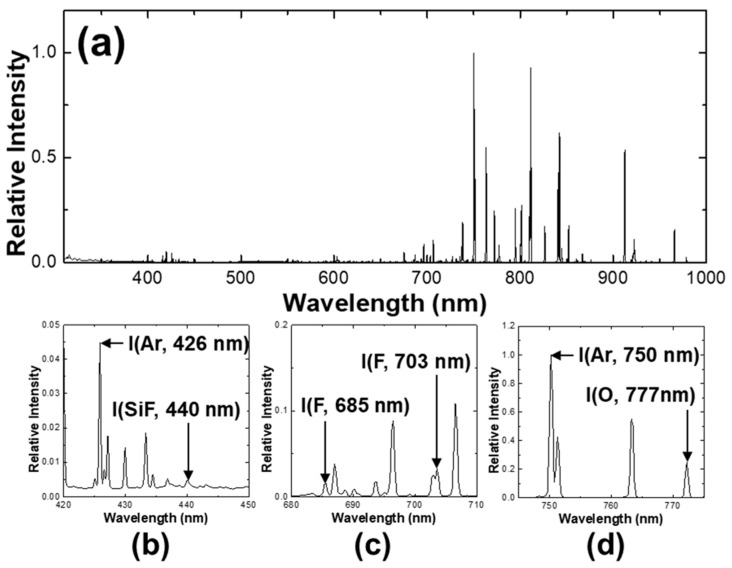
(**a**) OES full spectrum taken from SF_6_/O_2_/Ar plasma (operating conditions: 120/75/50 sccm, top main power 60 MHz 500 W, Btm bias power 2 MHz 150 W); (**b**) 426 nm of Ar emission and 440 nm of SiF emission; (**c**) 685 and 703 nm of F emission; (**d**) 750 nm of Ar emission and 777 nm of O emission. Details of each optical emission signal are described in Table A1 and Table A2 of Appendix A.

Figure 3 is an example of SF_6_/O_2_/Ar CCP OES spectrum. The OES spectrum observed with the spectrometer when SF_6_/O_2_/Ar flows into the chamber of 120/75/50 sccm, respectively, and the main power of top 60 MHz 500 W and bias power of bottom (Btm) 2 MHz 150 W were applied. A strong argon optical signal can be observed at 700 to 900 nm, and also the F and O atom optical signals are observed. F and O atoms are produced by the electron impact dissociation reaction of SF_6_ and O_2_. 685 and 703 nm of F atom emission is selected as shown in Figure 3c. In addition, 750 nm of Ar atom emission and 777 nm of O atom emission is selected as shown in Figure 3d. The 400 to 500 nm region shows a relatively weak intensity compared to the 700 to 900 nm region. Here, 426 nm of Ar emission and 440 nm of SiF emission can be observed as shown in Figure 3b. SiF is mainly produced through the electron impact dissociation reaction of SiF_4_ and SiF_2_. SiF_4_ is produced from four fluorine atoms combined with surface Si atom, which is a volatile product and it represents the chemical etching. SiF_2_ is desorbed by the impinging ion while surface Si atom is combined with two fluorine atoms, which results from the ion-enhanced etching. The amount of these by-products is related to the amount of etched Si, such as the amount of SiF increases while the amount of SiF_4_ and SiF_2_ increase. Therefore, the stronger SiF optical emission intensity represents the higher Si etch rate and the larger amount of etch by-product. Details of optical emission spectrum of Ar, F, O, and SiF are shown in Appendix A.

#### 2.3.1. Single Line Emission Intensity of Neutrals (PI_Single Line_)

PI_Single Line_ represents the single line emission intensity of specific species. If the upper level of the emission line is populated only from the ground state and ignores the transition from other excited states (corona model), the emission line intensity can be expressed as following Equation (1) [17]:

(1) $$I_{\lambda (pk)}^{X}=\Gamma_{pk}^{eff}n_{e}n_{X,g}<\sigma_{exc}v>=\Gamma_{pk}^{eff}n_{e}n_{X,g}\sqrt{\frac{2}{m_{e}}}\int_{0}^{\infty }\sigma_{exc}(\varepsilon )f(\varepsilon )\sqrt{\varepsilon }d\varepsilon$$

where $I_{\lambda (pk)}^{X}$ is the emission intensity of wavelength λ(pk) emitted when transiting to the *p*→*k* energy level of species *X*, $\Gamma_{pk}^{eff}$ is the branching ratio falling from the upper level *p* state to the *k* state among lower levels, $n_{e}$ is the electron density, $n_{X,g}$ is the ground state of X density, $\sigma_{exc}$ is the electron-impact excitation cross section of ground → *p* state, v is the electron velocity, $m_{e}$ is the mass of electron, and f(ε) is the electron energy distribution function. The single line emission intensity is proportional to the product of electron density and *X* species density. Thus, PI_Single Line_ represents the product of electron density and neutral density under operating conditions where the variation of electron temperature is assumed as small enough to be neglected.

**Figure 4:**
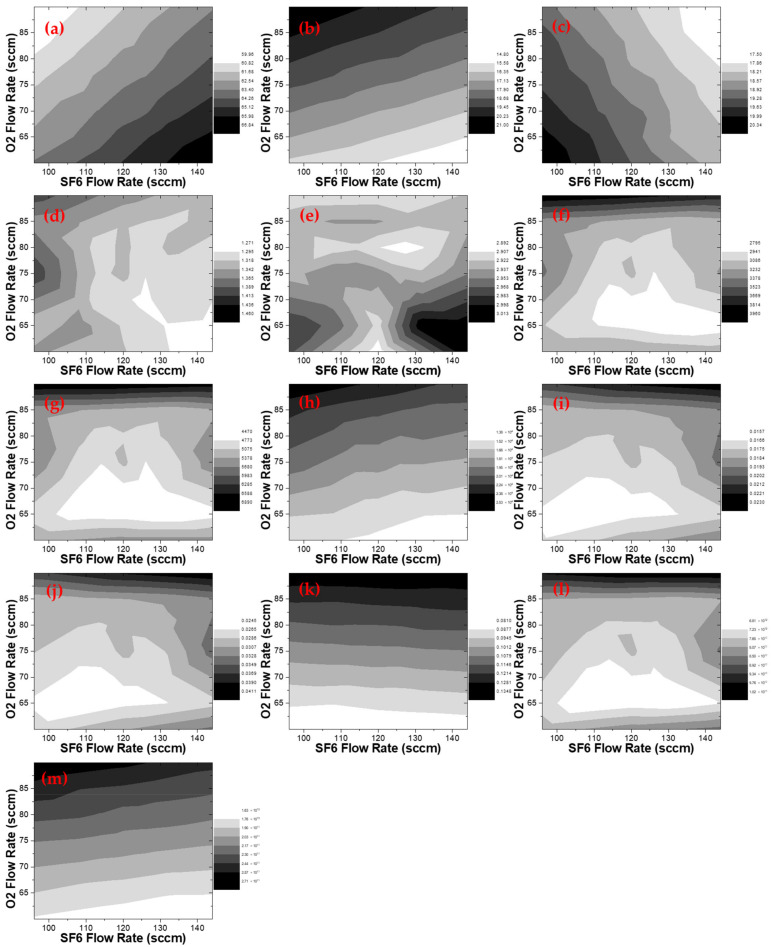
Plasma information variables with varying SF_6_ and O_2_ flow rates. The error of PI-OES is within 0.3% in one etch process. (**a**) Molar fraction of SF_6_ [%]; (**b**) molar fraction of O_2_ [%]; (**c**) molar fraction of Ar [%]; (**d**) ne [a.u.]; (**e**) Te [eV]; (**f**) I(F, 685 nm) [a.u.]; (**g**) I(F, 703 nm) [a.u.]; (**h**) I(O, 777 nm) [a.u.]; (**i**) I(F, 685 nm)/I(Ar, 750.4 nm) [a.u.]; (**j**) I(F, 703 nm)/I(Ar, 750.4 nm) [a.u.]; (**k**) I(O, 777 nm)/I(Ar, 750.4 nm) [a.u.]; (**l**) nF [cm^−3^]; (**m**) nO [cm^−3^].

Figure 4f–h shows the change of PI_Single Line_ according to the flow rate of SF_6_ and O_2_. Figure 4f,g shows the trend of I(F, 685 nm) and I(F, 703 nm). These variables decrease up to 40% as the SF_6_ increases and O_2_ decreases, since the F generation through the electron-impact dissociation reaction and SF_x_-O reaction decreases. In Figure 4h, I(O, 777 nm) increases nearly twice as the O_2_ flow rate increases. It implies that the electron-impact dissociation reaction between the electrons and O increases as the O_2_ flow rate increases.

#### 2.3.2. Electron Temperature (PI_Te_)

The electron temperature indicates the average energy of electrons in the plasma. As the electron temperature increases, the number of electrons causing the inelastic collision increases, and as a result, the rate of dissociation, excitation, and ionization reaction increase. Thus the electron temperature is an important variable to trace the variation the radical and ion production rate. In this study, the electron temperature is measured using the intensity ratio of Ar 425.9 nm and Ar 750.4 nm emission with the Maxwellian EEDF assumption [19]. The line ratio indicates the excitation temperature of electrons with higher energy than the excitation threshold energy. And the bulk electron temperature is assumed as the electron excitation temperature. The upper level energy difference between the emission lines, which equals to the difference of excitation threshold energy, is about 1.3 eV. For this reason, the ratio of excitation rate of the two upper levels have high sensitivity to the electron temperature, so that the variable of electron temperature, PI_Te_, can be obtained from the ratio of corresponding lines. It is expressed as following Equation (2):

(2) $$\frac{I_{425.9nm}^{Ar}}{I_{750.4nm}^{Ar}}=\frac{\Gamma_{425.9nm}^{eff}n_{e}n_{Ar,g}<\sigma_{exc,425.9nm}v>}{\Gamma_{750.4nm}^{eff}n_{e}n_{Ar,g}<\sigma_{exc,750.4nm}v>}=\frac{\Gamma_{425.9nm}^{eff}}{\Gamma_{750.4nm}^{eff}}\frac{<\sigma_{exc,425.9nm}v>}{<\sigma_{exc,750.4nm}v>}$$

Figure 4e shows PI_Te_ according to the change of SF_6_ and O_2_ flow rate, and it has a maximum variation of 2%. Since the variation of electron temperature is small enough, it is assumed to be a constant in the remainder of analysis.

#### 2.3.3. Neutral Density Ratio (PI_Density Ratio_)

The PI_Density Ratio_ represents the density ratio of neutral species. The density ratio between two neutral species is proportional to the emission line ratio of the species. With adoption of the corona model and a constant electron temperature, the relation of line ratio and neutral density ratio can be expressed as Equation (3) [20]:

(3) $$\frac{I_{\lambda (ab)}^{Y}}{I_{\lambda (cd)}^{X}}=\frac{\Gamma_{ab}^{eff}n_{e}n_{Y,g}<\sigma_{exc,ga}v>}{\Gamma_{cd}^{eff}n_{e}n_{X,g}<\sigma_{exc,gc}v>}=\frac{1}{C_{X}^{Y}}\frac{n_{Y,g}}{n_{X,g}}$$

In Equation (2), since the electron density term disappears and the reaction rate term can be treated as a constant under the constant electron temperature, the neutral density ratio can be the calculated product of constant actinometric coefficient $C_{X}^{Y}$ and line ratio $\frac{I_{\lambda (ab)}^{Y}}{I_{\lambda (cd)}^{X}}$. It is important to select the emission lines that satisfy the assumptions. The used line ratio is written down in Table 2 and actinometric coefficients have been cited in Lopaev et al., (2017) [20]. The change in the ratio of the optical emission intensities of F, O, and SiF to the Ar optical emission were observed. As the SF_6_ and O_2_ flow rates change, the Ar density can be monitored by the method introduced in a later Section 2.3.4. Using the line ratio and the density of Ar, the absolute density of the radicals of F and O can be calculated as shown in a later Section 2.3.5.

A variation of PI_Density Ratio_ is summarized in Figure 4i–k. Specifically, Figure 4k shows the density ratio of O and Ar, reveals that the density ratio increases significantly as the O_2_ flow rate increases.

#### 2.3.4. Inlet Gas Density (PI_Inlet Gas_)

PI_Inlet Gas_ is a variable indicating the density of the inlet gas species (SF_6_, O_2_, and Ar). It is difficult to find out the absolute density of the gas species in the chamber from the flow rates because the molar ratio of each gas varies with the conductance depending on the mass of the gas and the geometry of the chamber [21]. The inlet gas densities are obtained through the following procedure after the etch process.

1. In the situation where x, y, z sccm of SF_6_, O_2_, Ar flow into the chamber at the same time, the TVP position is fixed at a % to maintain the chamber pressure of 20 mTorr.
2. In the situation where the TVP Position is fixed at a % and only x sccm of SF_6_ flows in, the chamber pressure P_SF6_ is measured.
3. Next process is also carried out for O_2_ and Ar.
4. The density of species X can be obtained through the following Equation (4):
  (4) $$n_{X}=7.072\times 10^{14}\frac{P_{X}}{P_{SF_{6}}+P_{O_{2}}+P_{Ar}}[cm^{-3}]\;(X=SF_{6},O_{2},and\;Ar)$$

Figure 4a–c shows the molar fractions of SF_6_, O_2_, and Ar according to the flow rates of SF_6_ and O_2_, respectively. The molar fraction of SF_6_ varies from 60% to 67%, and O_2_ varies from 15% to 21%. Accordingly, Ar with the fixed flow rate fluctuates from 17.5% to 20%.

#### 2.3.5. Radical Density (PI_Radical_)

PI_Radical_ indicates the generated radical density. Radicals are generated from the electron-impact dissociation reaction of inlet gas such as SF_6_ and O_2_, and the radicals participate in the etching process directly so they are key parameters in the etch profile monitoring. It is difficult to obtain the absolute density of the desired species when the reference species density (Ar) is in an uncertain situation. However, as mentioned in Section 2.3.4., the argon density can be obtained after the etch process. In addition, the absolute density of radicals can be achieved using the argon density and line ratio from the OES Sensor. The Equation (5) is expressed as follows:

(5) $$n_{X,g}=C_{Ar}^{X}\frac{I_{\lambda (ab)}^{X}}{I_{750.4nm}^{Ar}}n_{Ar}$$

Figure 4k,l shows the density of F and O, respectively, which is PI_Radical_. F has a variation in the range of approximately 7 × 10^12^ to 1 × 10^13^ cm^−3^, and O has a variation in the range of 1.6 × 10^13^ to 2.7 × 10^13^ cm^−3^.

#### 2.3.6. Electron Density (PI_ne_)

PI_ne_ is a variable indicating the electron density. The electron is one of the most important particles in the plasma because it determines the reaction such as ion generating ionization, radical generating dissociation, and excitation reaction to emit optical signals. It can be calculated from the 750.4 nm emission line intensity and argon density. It can be used under the constant electron temperature which is obtained previously, and PI_ne_ represents a relative electron density value. The Equation (6) is expressed as follows:

(6) $$n_{e}=\frac{I_{750.4nm}^{Ar}}{n_{Ar}<\sigma_{exc}v>}$$

A variation of electron density can be found in Figure 4d. As SF_6_ increases, the electron density decreases up to 15% with an increase in the electron attachment reaction.

### 2.4. Etch Profile Quantification: Etch Depth, Bowing CD, Etch Depth Times Bowing CD (Rectangular Model), and Etch Area (Non-Rectangular Model)

**Figure 5:**
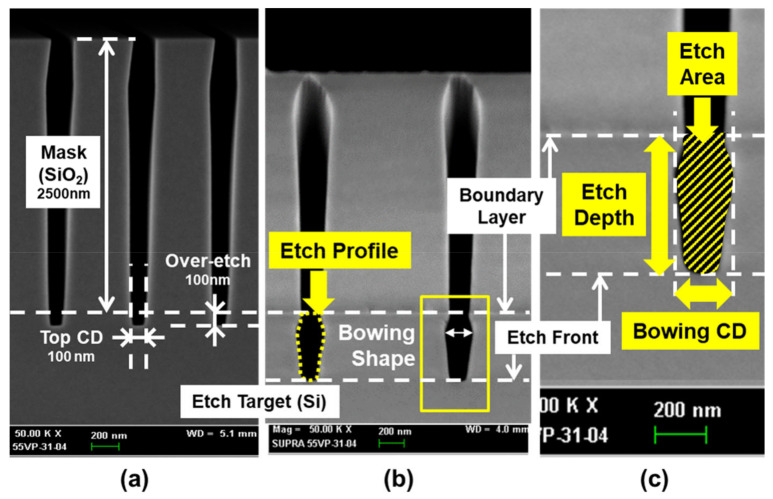
Definition of Si etch profile which is taken from a cross-sectional SEM image of Si with the SiO_2_ mask. Etch depth, bowing CD, etch area of trench, and etch profile are defined. (**a**) SEM image before the Si etch process with the etched SiO_2_ mask. (**b**) SEM image after the Si etch process. Yellow dotted line indicates etch profile. (**c**) Quantification of etch profile. Vertical position of etch front and SiO_2_/Si layer boundary are marked with white horizontal dashed lines. Etch depth, bowing CD, and etch area are marked with yellow box.

Figure 5 shows the procedure of quantifying the Si etch profile after the etch process from the cross-sectional SEM image. Figure 5a is a SEM image of the wafer before the etch. Hard mask SiO_2_ is on top of the Si layer and it has a trench etch profile with a thickness of 2500 nm and CD of 100 nm as shown in Figure 5a. When the etch process is conducted, the Si layer etched SEM image and etch profile is shown as Figure 5b. In Figure 5c, the definition of the etch depth, bowing CD, and etch area selected as targets for virtual metrology are described. The vertical position of the etch front is defined as the frontline of the etch profile. The SiO_2_-Si layer boundary is determined by the position of brightness difference that occurred in the SEM image. The etch depth is defined as the distance between the layer boundary and etch front. The bowing CD corresponds to the maximum width in the lateral direction of the etch profile under the layer boundary. The etch area corresponds to the shaded area of the etch profile under the layer boundary. MATLAB software was used to determine the boundary between the etched and non-etched area and calculate the etch area (non-rectangular model) inside the boundary. Here, assuming that the etch profile below the boundary layer is in a rectangular shape (rectangular model) with a width of bowing CD and a height of etch depth, the area of rectangle is the product of etch depth and bowing CD. The virtual metrology on the area of rectangular model (abbreviated to ED × BCD) and non-rectangular model (etch area) was also carried out. The etch depth and bowing CD are related to the vertical and lateral etch rates, respectively. The vertical etch rate is related to the amount of etchant reaching at the etch front and the amount of reaching ions participating in the ion-enhanced etch. The lateral etch rate is determined by the amount of etchant reaching the sidewall, the amount of forming a passivation layer, and the number of ions reaching the sidewall directly or after being refracted at the mask sidewall. In the case of etch area, it is related to the influx of etchants and ions since it corresponds to the total amount of etch reaction rate during the process caused by etchants and ions incident in the etch profile. They are main features composing the etch profile and PI-VM to predict the etch profile is developed in terms of four etch profile values (etch depth, bowing CD, ED × BCD, and etch area) by using the obtained EES, OES, and PI variables and the selected statistical analysis method.

## 3. Results and Discussion

### 3.1. Improvement of VM Performance with Plasma Information Variables

Table 3 summarized the R^2^ and features of PI-VM (etch depth) by sequentially including EES, PI-OES, and PI_Density_ in the input data set. A total of five PI-VM (etch depth) models based on statistics of MLR-SVS (refer to Introduction Session) were developed using five input data sets by including the PI variables. The input data of the first PI-VM model include the MFC flow rate and TVP position among EES data without PI variables. The second model includes matcher data with the first model input data to evaluate the effect of matcher data. The third and fourth models add PI_Single Line_ and PI_Line Ratio_ of PI-OES, respectively, for the purpose of evaluating the effect of PI-OES on the VM performance. In these PI-VM models, the effectiveness of PI_Single Line_ and PI_Line Ratio_ on the prediction accuracy is discussed. The fifth model demonstrates the improvement of accuracy of the VM when using PI_Density_ with EES and PI-OES.

In the first model, O_2_ flow rate, SF_6_ flow rate, and TVP position (On) are selected as features, and the prediction accuracy is R^2^ of 0.806. In the second model, when the information of matcher data is included, three factors related to LF (LF V mag., LF tune position, and LF load position) are selected and added to the features of the first model. The improvement of R^2^ from 0.806 to 0.819 is observed. It implies that the etch rate is mainly determined by the LF (bias power) rather than the VHF (main power) since the etch process is sensitive to the incident ion energy on the wafer.

The third model including PI_Single Line_ selects the O line emission as a feature rather than using the O_2_ flow rate when compared to the first model, and R^2^ slightly increases from 0.806 to 0.816. It implies that the O line emission, which contains the information of O and electron densities, increased R^2^ of the VM. When compared to the second model, the four variables are replaced to only one variable (I(O)). In spite that R^2^ decreased slightly, the number of features is reduced because the data from the OES is more effective to describe the process result rather than EES data.

The fourth model including PI_Line Ratio_ shows a prediction accuracy R^2^ of 0.835, and features of I(O)/I(Ar), I(F)/I(Ar), and I(SiF)/I(Ar) are additionally selected compared to the first model. Compared to the third model including PI_Single Line_, the prediction accuracy increased through containing more variables related to the etch profile rather than including one variable I(O).

The fifth model, including PI_Density_, shows the highest prediction accuracy R^2^ of 0.849. The O_2_ density, electron density of PI_Density_, I(F), I(O), I(SiF) of PI_Single Line_, and I(Ar)/I(F) of PI_Line Ratio_ are selected as features. By including PI_Density_, it is notable that all of the EES data are extracted and the prediction accuracy is significantly increased compared to the other models.

It is investigated that the role of the features on the improvement of VM accuracy. Firstly, it is considered that the role of the O_2_ density and the electron density of PI_Density_ correspond to species generate the primary etchant generation. O_2_ density can provide more information on the reaction rates directly rather than the flow rate. The O_2_ density only can be estimated by considering all of the information such as the flow rate of the other gas species, pumping speed, TVP position, operating pressure, and geometry of chamber. Thus, the O_2_ density is a more effective variable than the O_2_ flow rate to improve performance of VM. Also, the electron density is the variable that directly affects most of the reactions in the etch plasma as mentioned previously. Secondly, the contribution of F line emission I(F) and O line emission I(O) on improvement of the VM is considered. In the MLR method which is chosen in this study, the arbitrary variables A and B are selected as features and then the prediction can be expressed by a linear combination of only A and B. In addition, the prediction can be improved by using the product of A and B, for the case of plasma assisted chemical reactions. In that case, the prediction accuracy is improved by a linear combination of A, B, and also A times B (A × B). Similarly, in this study, prediction accuracy of etch depth PI-VM can be improved by using A × B rather than using only linear combination of A and B. Here, the F line emission and the O line emission are proportional to the product of the F and O density times and the electron density, respectively. Since O and F radicals are generated through the electron-impact dissociation reaction rate, they are proportional to the product of the electron density and inlet gas (SF_6_ and O_2_) densities. Therefore, it can be deduced that the PI-VM (etch depth) performance improved by including second step features corresponding to the product of the features as described in the first step, electron density and O_2_ density. For the third PI, the I(Ar)/I(F) of PI_Line Ratio_ reveals the VM development procedure more specifically. The etch rate can be classified into four basic plasma etch processes: Sputtering, pure chemical etching, ion-enhanced energy-driven etching, and ion-enhanced inhibitor etching [11]. Pure chemical etching refers to the etch reaction that occurs by reactive gas-phase etchant atoms or molecules. On the other hand, ion-enhanced energy-driven etching is an etch mechanism in which energetic ions are incident on the surface while supplying a neutral etchant such as F atoms, and it results a much faster etch rate than pure chemical etching or sputtering [22]. In the case of I(Ar), it is proportional to the product of Ar and electron density, which is proportional to the density of Ar ions. Therefore, it can be said that the I(Ar)/I(F) sensitively indicates the ion-enhanced energy-driven etching which is caused by positive ions. The fourth feature is I(SiF) which indicates the amount of etch process by-product. Si reacts with two or four F atoms, and it is removed from the wafer in the form of SiF_2_ or SiF_4_. Thus, I(SiF) is selected as a feature that represents the etch reaction rate and amount of the etch by-product from the etch profile.

### 3.2. Etch Profile PI-VM Models: Etch Depth/Bowing CD/ED × BCD (Rectangular Model)/Etch Area (Non-Rectangular Model)

Table 4 shows the prediction accuracy and features of the developed PI-VM models for etch depth, bowing CD, etch depth times bowing CD, and etch area. The first column of the table shows the results of PI-VM model for the etch depth explained in the prior section. As shown in second column, the PI-VM model predicting the bowing CD by MLR using the features of etch depth PI-VM shows a prediction accuracy R^2^ of 0.907, which is higher than PI-VM (etch depth). It can be explained that more accurate PI-VM was made on the bowing CD because PI-VM models had been developed only using the chemical composition variation, not the ion energy which dominates on the etch depth. When bowing CD is predicted with MLR-SVS using the data of EES, PI-OES, and PI_Density_, R^2^ is 0.93, showing the highest prediction accuracy. A total of 11 variables are selected by using SVS method. Those features and estimations are summarized in Table 4.

**Figure 6:**
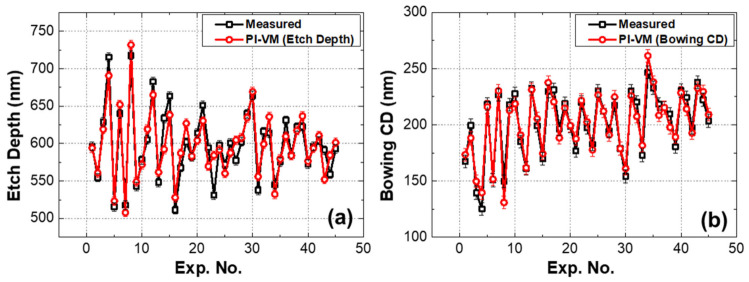
Comparison between the measured values (black) and PI-VM prediction values (red) (**a**) measured and PI-VM prediction values of etch depth (**b**) measured and PI-VM prediction values of bowing CD. The error of each value is 5.7 nm, which is the pixel size of the SEM image.

The Figure 6 shows the comparison of PI-VM (etch depth) and PI-VM (bowing CD) to the measurement results from SEM image of 45 wafers. Both models show high prediction accuracy with higher R^2^ than 0.8.

**Figure 7:**
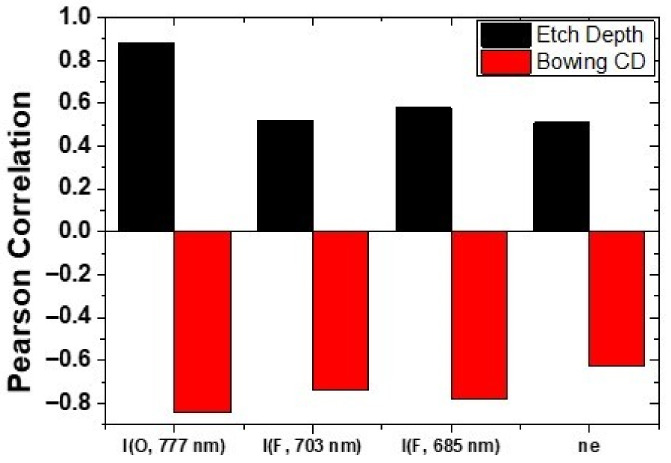
Pearson correlation coefficients of features commonly selected in the etch depth and bowing CD PI-VM for etch depth and bowing CD. Commonly selected features: I(O, 777 nm), I(F, 703 nm), I(F, 685 nm), and ne.

Figure 7 shows the Pearson correlation coefficients of features commonly selected in the PI-VM for etch depth and bowing CD. The Pearson correlation coefficient is a factor which represents the linear relationship between the two variables and it is calculated by dividing the covariance of two variables by the standard deviations of each variable. It has a value between −1 and 1, where the positive value means that one variable increases as the other variable increases, and vice versa. I(O), I(F), and electron density have a positive correlation for etch depth and a negative correlation for bowing CD. In the case of etch depth, the increase of O with electron density induces larger reaction rates of F radical generating O-SF_x_ (x = 1~5) reaction. Accordingly, the amount of F reaching the etch front increases with the etch depth [23]. On the other hand, in the case of bowing CD, the increased O with the increasing electron density forms more SiOF, which makes a role of passivation layer on the sidewall of etch profile [24]. For this reason, as O increases, the lateral etch rate and bowing CD decreases.

**Figure 8:**
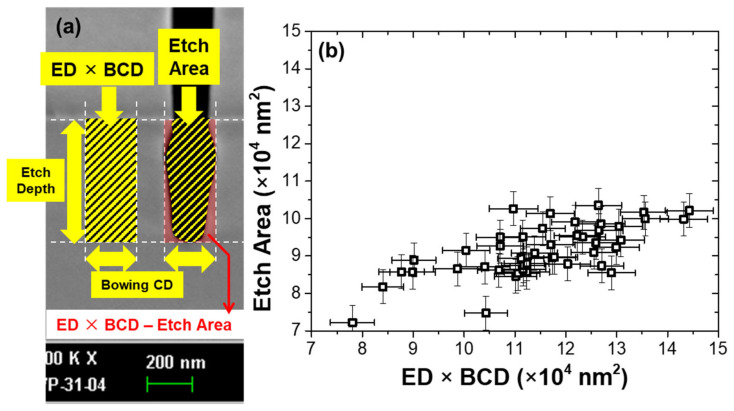
(**a**) Comparison diagram of ED × BCD and the etch area. Area of left striped rectangular region is ED × BCD (rectangular model). Area of right striped region is the etch area (non-rectangular model). The red area represents the difference between ED × BCD and the etch area. (**b**) Correlation between ED × BCD and the etch area. They show a low correlation R^2^ of 0.25.

Figure 8a,b shows the difference between ED × BCD and the etch area. In general, if R^2^ is higher than 0.8, it means that the VM model is reliable which closely predicts the actual value. However, in the case of PI-VM for ED × BCD and the etch area, despite the 11 variables selected to describe the target value, R^2^ is less than 0.8. In the case of ED × BCD, although R^2^ is close to 0.8, the etch profile value lacks physical meaning and it is far from the real etch area. The etch profile shows a bowing shape with a convex sidewall in this study. Otherwise, it is known to have a tapered etch profile that narrows downward in the high aspect ratio etch process. Moreover, the low correlation between ED × BCD and the etch area (R^2^ of 0.25) is shown in Figure 8b. This is due to the difference between ED × BCD and the etch area (red area in Figure 8a) is changing irregularly with the etch profile of the bowing shape. Thus, ED × BCD is only valid for the rectangular shape of etch profile and it is limited to representing the real etch area.

The rectangular etch area model can better explain the etch profile. The etch area can be analyzed as the total amount of Si removed by the etch reaction with incident F radicals and energetic ions into the etch profile during the process time. Simply, it is related to the incident flux of etchants and reaction rate at the surface of etch profile. Although the PI-VM (etch area) model is predicted with a low accuracy R^2^ of 0.5, it is significant in that the new target of virtual metrology is proposed for the purpose of predicting etch profile. It is expected that the prediction accuracy of VM can be improved by producing more features and PI variables sensitive to etch area. By developing a model that can accurately predict the etch area, it would become a cornerstone of predicting the etch profile beyond the perspective of etch depth and bowing CD.

## 4. Conclusions

The etch profile PI-VM, which can predict the etch profile in two-dimensions, has been developed using PI variables with EES and sensor data. It was demonstrated that the prediction accuracy of PI-VM improved by including PI-OES and PI_Density_ as input data. Since PI variables increased the accuracy of VM effectively rather than using the raw sensor data, it has been revealed that PI variables are essential to develop more precise and rapid process monitoring technology. It was shown that densities of inlet gas, radicals, and electron obtained by the sensor technology are especially important to develop precise VM among the PI variables. In order to develop PI-VM (etch profile), we have proposed the etch profile model that consisted of vertical and horizontal direction and four of etch profile quantified values: Etch depth, bowing CD, etch depth times bowing CD (rectangular model), and etch area (non-rectangular model). PI-VM (etch depth) and PI-VM (bowing CD) demonstrated high accuracy of etch profile monitoring models and were sensitive to etching and passivation induced by ions and radicals. PI-VM (ED × BCD) showed little lower R^2^ than PI-VM (etch depth) and PI-VM (bowing CD), implying that the reliable etch profile VM can be developed based on a rectangular model. Although PI-VM (etch area) showed the prediction accuracy R^2^ under 0.5, it is significant that physically meaningful monitoring target is newly presented. Furthermore, this implies that more accurate and reliable PI-VM (etch area) can be developed with the more information of radical and the ion behavior along the etch profile. This research showed that more sophisticated PI-VM (etch profile) can be developed with the PI variables which can trace the behavior of radical and ion in the etch profile. It will contribute to the advancement of technology for end point detection and etch profile control.

## Figures and Tables

**Table 1:** Etch process conditions to acquire the data for PI-VM for the etch profile (PI-VM (etch profile)) development.

Condition	Values
Pressure	20 mTorr
Top Power (60 MHz)	500 W
Btm Power (2 MHz)	150 W
Ar Flow Rate	50 sccm
SF_6_ Flow Rate	96–144 sccm
O_2_ Flow Rate	60–90 sccm
Process Time	300 s

**Table 2:** Description of PI-VM input data. It is divided into EES, PI-OES, and PI_Density_. EES consists of MFC flow rate, TVP position, and matcher cap position. PI-OES consists of PI_Single Line_ and PI_Line Ratio_, and PI_Line Ratio_ is divided into PI_Te_ and PI_Density Ratio_. PI_Density_ is divided into PI_Inlet Gas_, PI_Radical_, and PI_ne_.

Category		Name	Simple Expression	Target	Physical Meaning	Line Information
EES	MFC	MFC Flow Rate	X Flow Rate	X = SF_6_, O_2_, Ar	Inlet Gas Flow Rate	-
	TVP	TVP Pos.	TVP Pos.(X)	X = On, Off, (On-Off)	Chamber Conductance	-
	Matcher	Matcher Cap Pos.	X Y Pos.	X = VHF, LF Y = Load, Tune	Load Impedance of Plasma	-
PI-OES	PI_Single Line_	PI_Single Line_	I(X) or I(X,WL)	X = Ar, F, O, SiF WL = wavelength of optical emission	Single Line Emission Intensity	Ar (750.4 nm, 425.9 nm)
						F (685.6 nm, 703.7 nm)
						O (777.2 nm)
						SiF (440 nm)
	PI_Line Ratio_	PI_Te_	Te	-	Electron Temperature	Ar (425.9 nm)/Ar (750.4 nm)
		PI_Density Ratio_	I(X)/I(Y)	X = O, F, SiF @ Y = Ar Y = O, F, SiF @ X = Ar	Neutral Density Ratio	F (685.6 nm, 703.7 nm)/Ar (750.4 nm)
						O (777.2 nm)/Ar (750.4 nm)
						SiF (440 nm)/Ar (750.4 nm)
PI_Density_		PI_Inlet Gas_	nX	X = SF_6_, O_2_, Ar	Inlet Gas Density	-
		PI_Radical_	nX	X=F, O	Radical Density (PI_Density Ratio_ x nAr)	-
		PI_ne_	ne	-	Electron Density	I(Ar, 750.4 nm)/nAr

**Table 3:** R^2^ and features of PI-VM (etch depth) by sequentially including EES, PI-OES, and PI_Density_ in the input data set.

VM Model	Model 1	Model 2	Model 3	Model 4	Model 5
Prediction and statistical feature selection method	MLR-SVS	MLR-SVS	MLR-SVS	MLR-SVS	MLR-SVS
Input Data	EES (MFC + TVP)	EES (MFC + TVP + Matcher)	EES + PI_Single Line_	EES + PI_Line Ratio_	EES + PI-OES + PI_Density_
R^2^	0.806	0.819	0.816	0.835	0.849
Features	O_2_ Flow Rate SF_6_ Flow Rate TVP Pos. (On)	O_2_ Flow Rate SF_6_ Flow Rate TVP Pos. (On) LF V Mag LF Tune Pos. LF Load Pos.	I(O) SF_6_ Flow Rate TVP Pos. (On)	O_2_ Flow Rate SF_6_ Flow Rate I(O)/I(Ar) TVP Pos. (On) I(F)/I(Ar) I(SiF)/I(Ar)	nO_2_ I(F) ne I(O) I(Ar)/I(F) I(SiF)

**Table 4:** Prediction accuracy and features of PI-VM models for etch depth, bowing CD, ED × BCD, and etch area.

Target	Etch Depth	Bowing CD	Bowing CD	Etch Depth Times Bowing CD	Etch Area
VM Model	MLR-	MLR	MLR-SVS	MLR-SVS	MLR-SVS
Input Data	EES + PI-OES + PI_Density_	Same features of PI-VM (Etch Depth)	EES + PI-OES + PI_Density_	EES + PI-OES + PI_Density_	EES + PI-OES + PI_Density_
R^2^	0.849	0.907	0.930	0.776	0.492
Features	nO_2_ I(F) ne I(O) I(Ar)/I(F) I(SiF)	nO_2_ I(F) ne I(O) I(Ar)/I(nF) I(SiF)	I(O) LF Tune Pos. nSF_6_ LF Load Pos. nAr SF_6_ Flow Rate ne I(F)/I(Ar) I(SiF)/I(Ar) VHF Tune Pos. I(Ar, 426)	I(F) I(SiF)/I(Ar) VHF Load Pos. TVP Pos. (On-Off) ne VHF Tune Pos. LF Load Pos. nO_2_ I(O)/I(F) I(F)/I(Ar) SF_6_ Flow Rate	I(O)/I(F) ne I(SiF) LF Load Pos. TVP Pos.(Off) I(F)/I(O) LF Tune Pos. Te LF V Mag. VHF Load Pos. VHF Tune Pos.

## Data Availability

The data presented in this study is available upon request from the corresponding author.

## Appendix A

**Table A1:** Optical emission spectrum information of argon, fluorine, and oxygen atom [25].

Species	WL [nm]	A-Rate [10^6^ s^-1^]	E_lower_ [eV]	E_upper_ [eV]	Lower Level			Upper Level		
					Conf.	Term	J	Conf.	Term	J
Ar	425.9	3.98	11.828	14.738	3s^2^3p^5^(^2^P_1/2_)4s	^2^[1/2]	1	3s^2^3p^5^(^2^P_1/2_)5p	^2^[1/2]	0
Ar	750.4	45	11.828	13.480	3s^2^3p^5^(^2^P_1/2_)4s	^2^[1/2]	1	3s^2^3p^5^(^2^P_1/2_)4p	^2^[1/2]	0
F	685.6	48.7	12.697	14.505	2s^2^2p^4^(^3^P)3s	^4^P	3/2	2s^2^2p^4^(^3^P)3p	^4^D	7/2
F	703.7	42.3	12.985	14.746	2s^2^2p^4^(^3^P)3s	^2^P	3/2	2s^2^2p^4^(^3^P)3p	^2^P	3/2
O	777.2	36.9	9.146	10.741	2s^2^2p^3^(^4^S)3s	^5^S	2	2s^2^2p^3^(^4^S)3p	^5^P	1

**Table A2:** Optical emission spectrum information and production reaction of silicon fluoride (SiF) [26].

Species	WL [nm]	E_lower_ [eV]	E_upper_ [eV]	Lower Level	Upper Level	SiF Production Reaction
SiF	440	0	2.814	X^2^Π_3/2_	A^2^Σ	^1a^ (R1-1) Si + 4F → SiF_4_ (R1-2) e + SiF_4_ → SiF + 3F + e ^1b^ (R2-1) X^+^ + Si:SiF_2_ → X + SiF_2_ (g) (R2-2) e + SiF_4_ → SiF_2_ + 2F + e (R2-3) e + SiF_2_ → SiF + F + e

^1a^ Surface reaction of silicon with fluorine atoms. SiF_4_ is a volatile product and produced through chemical etch. ^1b^ Surface reaction of silicon with fluorine atoms. SiF_2_ is mainly produced through ion impingement, while surface Si is combined with two of F atoms, which is called ion-enhanced etching.
